# Comparative Analysis of Twenty Intraocular Lens Power Calculation Formulas in Medium-Long Eyes

**DOI:** 10.3390/diagnostics16142281

**Published:** 2026-07-21

**Authors:** Wiktor Stopyra, Oleksiy Voytsekhivskyy, Andrzej Grzybowski

**Affiliations:** 1 MW-Med Eye Center, 31-416 Krakow, Poland; 2 Kyiv Clinical Ophthalmology Hospital Eye Microsurgery Center, 03680 Kyiv, Ukraine; iolcalculation@gmail.com; 3 Institute for Research in Ophthalmology, Foundation for Ophthalmology Development, 60-836 Poznan, Poland; a.grzybowski@uwm.edu.pl; 4 Department of Ophthalmology, University of Warmia and Mazury in Olsztyn, 10-719 Olsztyn, Poland

**Keywords:** cataract, medium-long eyes, intraocular lenses, SRK/T formula

## Abstract

**Background/Objectives**: The choice of appropriate intraocular lens (IOL) power calculation formulas depends on the axial length of the eye. This study compares the accuracy of twenty formulas in medium-long eyes (24.50–25.99 mm). **Methods**: Data of patients with medium-long eyes, who underwent uneventful phacoemulsification between January 2018 and September 2023, were retrospectively reviewed. Preoperative IOL power was calculated using the IOLMaster 700 with six formulas: Barrett Universal II, Haigis, Hoffer Q, Holladay 1, Holladay 2, and SRK/T. Three months postoperatively, refraction was measured. Postoperative IOL power calculations were then performed using fourteen additional formulas: Castrop, EVO 2.0, Hoffer QST, K6, Kane, Karmona, Ladas Super Formula AI (LSF AI), Naeser 2, Olsen (OLCR), Olsen (standalone), PEARL-DGS, T2, VRF CMAL, and VRF-G. The main outcome measures included standard deviation (SD) of the prediction error (PE) and the percentage of eyes with PE within ±0.25 D, ±0.50 D, ±0.75 D, and ±1.00 D. **Results**: Ninety-five eyes with axial lengths ranging from 24.52 mm to 25.97 mm were included. SD values among the twenty formulas ranged from 0.179 (SRK/T) to 0.468 (Olsen OLCR). The percentage of eyes with PE within ±0.50 D ranged from 75.79% (Olsen OLCR) to 98.95% (SRK/T). The SRK/T formula, followed by Holladay 1, demonstrated significantly higher accuracy than most other formulas, while Olsen (OLCR) and Castrop were the least accurate. **Conclusions**: SRK/T provided the highest accuracy in medium-long eyes, with Holladay 1 performing similarly well. All evaluated formulas achieved PE within ±0.50 D in over 74% of cases.

## 1. Introduction

Cataract surgery is the most frequently performed ophthalmic procedure worldwide, and its success is increasingly defined not only by the restoration of transparency but also by the achievement of optimal refractive outcomes [1]. Patients today expect spectacle independence, which requires highly precise intraocular lens (IOL) power calculation [2]. While improvements in optical biometry and surgical techniques have markedly reduced sources of error, predicting the postoperative refractive result remains a challenge [3]. This is primarily due to the fact that the effective lens position (ELP), the most important factor influencing the refractive power of the implanted lens, cannot be measured directly and must instead be estimated mathematically [4].

To address this limitation, numerous IOL power calculation formulas have been introduced over the last decades [5,6,7,8,9,10,11]. These can be broadly categorized into regression-based, vergence-based, ray-tracing, hybrid, and artificial intelligence (AI)-based models [12]. Each approach offers theoretical advantages, and many formulas demonstrate excellent accuracy in eyes of average axial length [13,14]. However, outcomes are often less predictable in eyes with atypical biometry, particularly at the shorter and longer ends of the spectrum [15,16,17,18,19].

The intermediate zone—commonly referred to as medium-long eyes, defined as axial length (AL) between 24.50 mm and 25.99 mm—has received far less attention in the literature [20,21]. This range is clinically relevant because different authors adopt varying thresholds for defining high myopia, which has led to inconsistent classification, and as a consequence, underrepresentation of medium-long eyes in comparative studies [22,23,24,25,26,27]. As a result, there is no consensus regarding which IOL formulas provide the most accurate refractive outcomes in this specific subgroup [28,29,30].

Assessing formula performance requires reliable statistical endpoints [31]. Traditionally, mean absolute error (MAE) and median absolute error (MedAE) have been reported [32], with some authors preferring root mean square absolute error (RMSAE) to account for the skewed distribution of refractive prediction errors [33]. Standard deviation (SD) of the prediction error, however, remains a robust indicator of formula precision and allows for a direct comparison across different models [27,34]. Another clinically meaningful benchmark is the percentage of eyes achieving a postoperative refraction within ±0.50 diopters (D) of the target, which directly reflects the likelihood of satisfactory functional vision for patients [35].

Given the growing number of available IOL calculation methods, there is a clear need for systematic comparisons that include both traditional and modern formulas, applied to well-defined biometric subgroups [36]. The present study addresses this gap by evaluating 20 contemporary IOL power calculation formulas specifically in medium-long eyes. Accuracy was assessed using the SD of the prediction error and the percentage of eyes with PE within ±0.50 D. To our knowledge, this is one of the few studies dedicated exclusively to this AL interval, and among the most comprehensive comparisons of formulas published to date [37].

## 2. Materials and Methods

We retrospectively evaluated 95 eyes of 95 patients with medium-long ALs, which underwent uneventful sutureless phacoemulsification and monofocal IOL implantation between January 2018 and September 2023. Only one eye of each patient was considered in the study, in accordance with Hoffer’s recommendations [32]. In patients who underwent bilateral cataract surgery (*n* = 11), only the right eye was enrolled in analysis. Inclusion criteria comprised an AL between 24.50 mm and 25.99 mm and Wisconsin grade 3 or 4 cataract [38]. Initially, 117 eyes were assessed for eligibility. Exclusion criteria were intraoperative (*n* = 2) or postoperative complications (*n* = 3), history of ocular surgery (*n* = 3), corneal pathology (*n* = 5), postoperative best corrected visual acuity (BCVA) below 0.8 (*n* = 8), and corneal astigmatism greater than 2.0 D (*n* = 1), resulting in the final cohort study of 95 eyes.

The study was conducted in accordance with the tenets of the Declaration of Helsinki and received approval from the Institutional Review Board of the Foundation for the Advancement of Ophthalmology “Ophthalmology 21” (approval number: 01/2024). Written informed consent was obtained from each patient prior to routine cataract surgery. The study is compliant with the Health Insurance Portability and Accountability Act, and all patient data were anonymized to ensure confidentiality.

Preoperative biometric data including AL, keratometry (K), anterior chamber depth (ACD), lens thickness (LT), and white-to-white corneal diameter (WTW) were obtained using the IOLMaster 700 (Carl Zeiss Meditec AG, Jena, Germany). Central corneal thickness (CCT) was measured with an auto kerato-refracto tonometer TRK-2P (Topcon Corporation, Tokyo, Japan). Postoperative manifest (non-cycloplegic) refraction performed by appropriately trained medical personnel was assessed three months after cataract surgery. The same technician, masked to the predicted refraction, conducted all examinations. The value of postoperative manifest refraction was measured at a distance of 6 m, as recommended by Simpson and Charman [39].

IOL power was calculated using 20 formulas, comprising both traditional and AI-based approaches. For each formula, optimized lens constants were applied to target zero mean prediction error (PE). The predicted postoperative refraction was compared with the actual spherical equivalent outcome, defined as the sum of the spherical value and half the cylindrical component. PE was defined as the difference between predicted and achieved refraction, with positive values indicating a hyperopic shift and negative values indicating a myopic error. Optimization of the Castrop, Hoffer Q, Holladay 1, Naeser 2, SRK/T, VRF CMAL, and VRF-G formulas was performed using the Goal Seek function in Microsoft Excel, as previously recommended [35]. The optimized ACD constants for the Olsen (standalone) formula (PhacoOptics software, version 1.10.100.2029; IOL Innovations ApS, Aarhus, Denmark) and the Olsen (OLCR) formula (version i8.0.0.0; Haag-Streit AG, Köniz, Switzerland) were 4.788 and 4.735, respectively. Optimization of the Holladay 2 formula was performed automatically using HIC.SOAP Pro software (version 2018.0425; Holladay Consulting, Inc., Bellaire, TX, USA). For the Hoffer QST and Karmona formulas, the Excel spreadsheets provided in the Research sections of their respective websites (https://HofferQST.com, https://karmona-iol.com) were used for constant optimization. For the Barrett, EVO 2.0, Cooke K6, Kane, LSF AI, and PEARL-DGS formulas, the optimized A-constant was determined empirically by iterative adjustment until a mean PE of zero was achieved. For each formula, the primary objective of the optimization process was to obtain an IOL constant yielding a mean PE as close to zero (0.000 D) as possible. This approach ensured equivalent conditions for the comparative analysis of all formulas. The optimized IOL constants were derived exclusively from the study dataset, were not cross-validated on an independent dataset, and were used only for the analyses reported in this study.

The primary outcome was the SD of PE. As secondary outcomes, we assessed the percentage of eyes achieving a PE within ±0.50 D.

### Statistical Analysis

All statistical analyses were performed using IBM SPSS Statistics for Windows, Version 22.0 (IBM Corp., Armonk, NY, USA) and R Project 4.3.0 for Statistical Computing (https://www.r-project.org/). The normality of PE distribution was tested with the Kolmogorov–Smirnov test. The primary outcome, the SD of PE, was compared across formulas using the heteroscedastic (HC) method with Holm sequential correction. Since the SD is related to the square value of the difference of each value from the mean, it is affected by outliers. However, we are interested in outliers, which are outcomes we want to avoid in IOL power calculations. Recently, Holladay proposed RMSAE as an alternative to SD for describing the distribution of PEs in subgroups such as long eyes, short eyes, and eyes after corneal refractive surgery, i.e., groups with non-zero PEs [33]. Since our study cohort included only medium-long eyes, which are relatively close to the standard AL range, and eyes with previous corneal refractive surgery were excluded, the choice of SD for the analysis appears to be the most appropriate. A *p*-value < 0.05 was considered statistically significant.

For the secondary outcome, pairwise comparisons of the percentage of eyes within ±0.50 D of PE were conducted using the McNemar Chi-squared test with continuity correction. The Holm method was applied to adjust *p*-values for multiple testing.

A minimum sample size of 77 eyes was estimated to provide 95% confidence that the true proportion lies within ±5% of the observed value (PS program, Version 3.0.12; Dupont WD, USA, 2012).

## 3. Results

A total of 95 eyes (40 from male and 55 from female patients) were included in the study. Only one eye from each participant was examined, in accordance with the Hoffer protocol [32]. The AL of the examined eyes ranged from 24.52 mm to 25.97 mm. Demographic characteristics of the participants and biometric data of the enrolled eyes are presented in Table 1.

**Figure 1:**
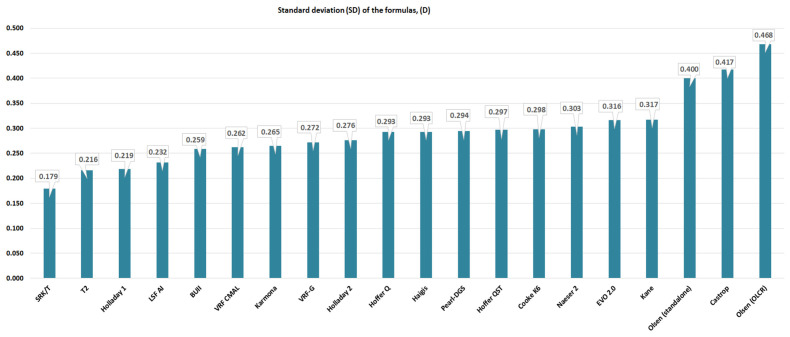
Standard deviation of the studied formulas. Formulas are ranked in ascending order.

The SD values of the twenty evaluated formulas ranged from 0.179 (SRK/T) to 0.468 (Olsen OLCR). Detailed results for all formulas are presented in Figure 1.

Statistical comparisons of SD were performed using the heteroscedastic method with Holm correction. A *p*-value < 0.05 was considered statistically significant. Exact *p*-values for each pairwise comparison of formulas are summarized in Table 2.

**Figure 2:**
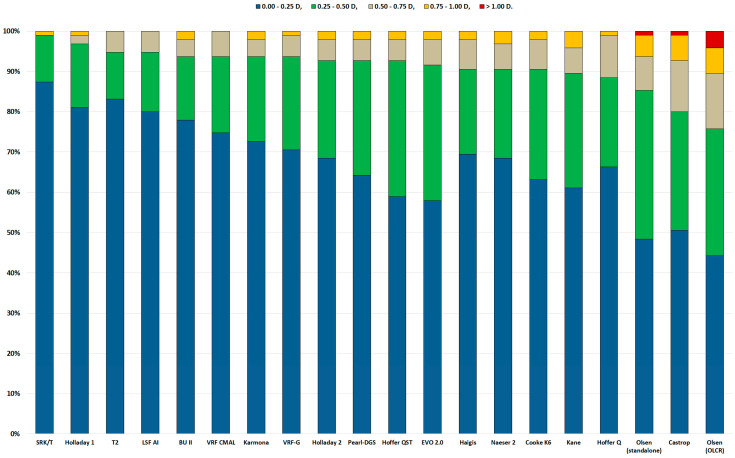
Percentage of eyes with prediction error (PE) within  ±0.25 D,  ±0.50 D,  ±0.75 D, and  ±1.00 D.

In turn, the percentage of eyes with a PE within ±0.50 D ranged from 75.79% (Olsen OLCR) to 98.95% (SRK/T), as shown in Figure 2.

The McNemar Chi-squared test with continuity correction was used to compare results between pairs of formulas. *p*-values < 0.05 were considered statistically significant, and the corresponding significant results for specific formula pairs are presented in Table 3.

SRK/T and Holladay 1 achieved a statistically higher percentage of eyes with PE within ±0.50 D compared with Olsen OLCR and Castrop. Meanwhile, Barrett Universal II, EVO 2.0, Hoffer QST, Holladay 2, Karmona, LSF AI, PEARL-DGS, T2, VRF CMAL, and VRF-G also demonstrated a statistically higher percentage of eyes with PE within ±0.50 D than Olsen OLCR.

Results of optimized constants and all refractive outcomes i.e., PE, SD, mean absolute deviation (MAD), median absolute error (MedAE), mean absolute error (MAE) as well as percentage of patients within PE ± 0.25 D, ±0.50 D, ±0.75 D and ±1.00 D for all studied formulas are summarized in Table 4.

## 4. Discussion

In the study, the SRK/T formula achieved the lowest SD value (0.179) and the highest percentage of eyes within 0.5 D of the target. Although SRK/T, as a third-generation formula, has been available for many years, it still provides clinically good results, as documented in numerous published articles.

Natung et al., in a study involving 219 eyes, reported that among the six formulas tested (including Barrett Universal II and Hill-RBF 3.0), the SRK/T formula yielded the lowest SD (0.2642) in eyes with an AL greater than 24.5 mm, which is consistent with our findings [40]. The higher SD observed in their study may be attributed to the inclusion of eyes without an upper limit of AL, whereas in our analysis, the maximum AL was restricted to 26.0 mm. It is well-established that in myopic eyes, the predictive accuracy of IOL power calculation formulas decreases with increasing AL [14,21,28]. Chen et al. estimated that each 1-mm increase in AL reduces the AE by approximately 0.1 D in eyes with AL > 26 mm, and by up to 1.1 D in extremely long eyes when AL > 33 mm [41]. However, the study by Natung et al. has several important limitations, including a relatively small number of long eyes (*n* = 45), the exclusion of eyes with a postoperative cylinder greater than 1 D, and the analysis of 219 eyes from 173 patients, which does not comply with Hoffer’s recommendations [32]. Despite these limitations, the study demonstrated that the SRK/T formula remains a reliable competitor to newer and widely used formulas such as Barrett Universal II and Hill-RBF 3.0 [40].

Another study published this year, similarly to ours, demonstrated high accuracy of the SRK/T formula in IOL power calculation. Eight formulas (SRK/T, Kane, Barrett Universal II, EVO 2.0, LSF AI, Haigis, Hoffer Q, and Holladay 1) were tested in 108 eyes, with SRK/T achieving the lowest SD of 1.16 D [42]. The SD value reported in that study differs substantially from ours, as the IOL power was calculated in pediatric eyes, which remains a significant clinical challenge [43,44]. Furthermore, the study conducted by Jin et al. has several important limitations, such as its retrospective nature, lack of effective lens position assessment, and the inclusion of 108 eyes from 83 patients, which does not comply with the protocol proposed by Hoffer et al. [32,42].

Malik et al., in a study comprising 100 eyes, demonstrated that the SRK/T formula is as reliable as Barrett Universal II (*p* = 0.078) and Hill-RBF 2.0 (*p* = 0.221) in IOL power calculation [45]. However, their methodology differed slightly, as they used the mean prediction error (MPE) as the primary outcome measure. Moreover, their study has several notable limitations, including a relatively small sample size, the evaluation of only three formulas, and the use of Hill-RBF 2.0, which is less accurate than the newer Hill-RBF 3.0 version [46].

Priji et al., in the study evaluating the accuracy of six formulas (Hill-RBF, Kane, Barrett Universal II, Hoffer Q, Holladay 1, and SRK/T), demonstrated that the SRK/T formula produced the lowest SD (0.13) in eyes with an AL exceeding 24.0 mm; however, the difference was not statistically significant [22]. Nevertheless, preoperative biometric data were obtained using an ultrasound immersion technique, which is less accurate than the swept-source optical coherence biometry available in devices such as the IOLMaster [47]. Another limitation of their study was the small sample size (*n* = 50).

Collectively, these studies, along with our current results, support the clinical reliability of the SRK/T formula in predicting IOL power, even when compared with newer, AI-assisted or theoretical formulas. Despite its age and simplicity, SRK/T continues to demonstrate competitive accuracy, particularly when used within the recommended biometric range and with modern optical measurement techniques.

The SRK/T formula, developed by Donald R. Sanders, John A. Retzlaff, and Manus C. Kraff—where “T” denotes *theoretical*—was introduced in 1990 [48]. This formula combines a theoretical model of the eye with a linear regression approach to improve the accuracy of IOL power prediction. By iteratively refining its parameters across five datasets comprising 1677 posterior chamber lens cases, the SRK/T formula optimizes estimates of postoperative ACD, retinal thickness, AL correction, and corneal refractive index. A key advantage of the SRK/T formula lies in its flexibility: it can be applied using empirically derived SRK A-constants accumulated over years of clinical use, or through the direct estimation of ACD. The primary variables incorporated into the SRK/T model are AL, K, and ACD.

Medium-to-long eyes were first distinguished by Kenneth Hoffer in his 2000 study, in which he assessed the accuracy of four formulas (SRK/T, Hoffer Q, Holladay 1, and Holladay 2) in 317 eyes, 52 of which had an AL between 24.5 mm and 26.0 mm [20]. Even at that time, the SRK/T formula demonstrated high accuracy in this subgroup, achieving the lowest standard deviation (SD = 0.401). However, only third- and fourth-generation formulas were evaluated in that study, and preoperative biometric data were obtained using the ultrasound immersion technique, which likely explains the relatively higher SD compared with that observed in our study (0.179) [47].

Another study that included medium-long eyes (*n* = 340) was conducted by Kane et al., who evaluated ten formulas across a total of 3122 eyes [21]. Barrett Universal II achieved the lowest MAE (0.333), while Holladay 1 produced the second-lowest MAE (0.374). Although heteroscedastic statistics were not applied in their analysis, the results were comparable to ours, where both Barrett Universal II and Holladay 1 showed some of the lowest SD values (0.259 D and 0.219 D, respectively). The study by Kane et al. had several limitations, including data collected from multiple surgeons, refraction measured by different personnel, and most importantly, the inability to fully optimize the IOL constants for some formulas such as Hill-RBF, LSF, and Barrett Universal II.

The accuracy of seven formulas in 70 medium-long eyes was analyzed by Voytsekhivskyy [10]. In his study, the Holladay 1 formula achieved the lowest standard deviation (SD = 0.260), followed closely by SRK/T (SD = 0.262). Although those results are comparable to ours, direct comparison between the studies is difficult, as the earlier analysis did not include several modern formulas—particularly those based on AI—which were not yet available at that time.

There are several limitations to this study. First, all patients were implanted with the same IOL model, which may limit the generalizability of the results to lenses of different optical designs. The IOLs evaluated in this study were anterior asymmetric biconvex, whereas other designs such as equi-biconvex are also commonly used. Differences in IOL geometry may influence the PE, and consequently, affect the relative performance of the tested formulas. However, there are two schools of thought regarding the optimal methodology for evaluating IOL power calculation formulas in clinical practice [20]. In this study, we adopted the approach that prioritizes standardization—using a single IOL model—so that the formulas themselves remain the only variable under investigation. This approach follows the recommendations proposed by Hoffer and Savini [35]. Second, ocular parameters such as K, ACD, and LT were not analyzed in relation to the prediction accuracy of the formulas. Some authors have reported systematic biases in prediction error when plotted against not only AL but also K, ACD, and LT, indicating that these factors may influence formula performance [26]. Third, pupil dilation was not considered. Although some studies have examined the impact of pupil dilation on the accuracy of IOL power calculation formulas, these investigations have been limited to a few formulas, including SRK/T, Haigis, and Barrett Universal II [49]. Fourth, patient age was not analyzed. Recent evidence suggests that the accuracy of IOL power calculation formulas, including AI-based models, may vary across different age groups, although most of these studies have focused on pediatric populations [50]. Finally, the absence of certain modern formulas represents another limitation. The Hill-RBF formula, which is a purely data-driven method, was not included. Several other recently introduced AI-based formulas (e.g., Nallasamy, Zhu-Lu, Zeiss AI) and ray-tracing methods (e.g., Okulix) were also excluded from the analysis. On the other hand, the inclusion of as many as 20 formulas constitutes a significant strength of the study.

## 5. Conclusions

The study demonstrates that the SRK/T formula provides highly accurate outcomes in medium-long eyes. Because it is integrated into many biometric measurement devices, the formula remains widely accessible and continues to be a practical option for IOL power calculation. In clinical practice, SRK/T serves as a reliable tool for obtaining relatively accurate results, particularly in settings where access to more advanced technologies may be limited, thereby retaining substantial clinical relevance.

Overall, all 20 formulas evaluated in this study achieved prediction PE within ±0.50 D in more than 74% of eyes, which is consistent with the benchmarks reported by the European Registry of Quality Outcomes. These findings reinforce the importance of both classic and modern formulas in contemporary cataract surgery and highlight the ongoing clinical value of SRK/T as a dependable reference standard. Further studies involving larger and more diverse populations, as well as the inclusion of advanced AI-based formulas, are warranted to validate and expand upon these results.

## Figures and Tables

**Table 1:** Demographics of the study subjects.

Demographics	Mean (±SD)	Range
Age	74.57 ± 9.08	52–94
Gender M/F, %	40/55	42.1%/57.9%
Axial Length (mm)	25.27 ± 0.41	24.52–25.97
Corneal Power, Km (D)	42.50 ± 0.91	40.30–44.10
Corneal Astigmatism Magnitude, Cyl (D)	0.66 ± 0.33	0.09–1.66
Anterior Chamber Depth (mm)	3.28 ± 0.43	2.38–4.38
Lens Thickness (mm)	4.17 ± 0.40	3.14–5.06
Corneal Diameter (mm)	12.43 ± 0.35	11.70–13.10
Central Corneal Thickness (mm)	0.558 ± 0.034	0.478–0.623
IOL Power (D)	17.35 ± 1.77	14.0–22.0

Km = average corneal power, Cyl = corneal astigmatism magnitude, SD = standard deviation of the error, D = diopter.

**Table 2:** Statistical comparison of the standard deviation values (SD) according to the heteroscedastic (HC) method of the 20 formulas for the medium-long (24.50–25.99 mm) eyes (the Holm correction was applied, and *p* values less than 0.05 were considered statistically significant). The formula from the first column exhibits the lower SD within the given pair. Statistically significant differences were found predominantly for the SRK/T (*p* < 0.05), Holladay 1 (*p* < 0.05), T2 (*p* < 0.05), Castrop (*p* < 0.05), Barrett (*p* < 0.05), LSF AI (*p* < 0.05), Olsen (standalone) (*p* < 0.05) and Olsen (OLCR) (*p* < 0.000) formulas.

Formulas *p* Value:	BUII	Castrop	EVO 2.0	Haigis	Hoffer Q	Hoffer QST	Holladay 1	Holladay 2	Cooke K6	Kane	Karmona	LSF AI	Naeser 2	Olsen (OLCR)	Olsen (Standalone)	PEARL-DGS	SRK/T	T2	VRF CMAL	VRF-G
HC-method																				
BUII	--	0.000 *	0.000 *	0.037 *	0.225	0.006 *		0.984	0.000 *	0.000 *	0.984		0.004 *	0.000 *	0.000 *	0.000 *			0.984	0.984
Castrop		--												0.000 *						
EVO 2.0			--							0.984				0.000 *	0.000 *					
Haigis		0.000 *	0.984	--		0.984			0.984	0.984			0.984	0.000 *	0.000 *	0.984				
Hoffer Q		0.003 *	0.984	0.984	--	0.984			0.984	0.984			0.984	0.000 *	0.015 *					
Hoffer QST		0.018 *	0.984			--			0.984	0.984			0.984	0.000 *	0.024 *					
Holladay 1	0.273	0.000 *	0.000 *	0.000 *	0.000 *	0.000 *	--	0.010 *	0.002 *	0.001 *	0.123	0.984	0.000 *	0.000 *	0.000 *	0.002 *			0.051	0.064
Holladay 2		0.000 *	0.011 *	0.871	0.984	0.704		--	0.984	0.012 *			0.058	0.000 *	0.000 *	0.984				
Cooke K6		0.000 *	0.307						--	0.759			0.984	0.000 *	0.000 *					
Kane		0.000 *								--			0.984	0.000 *	0.000 *					
Karmona		0.000 *	0.014 *	0.984	0.984	0.308		0.984	0.518	0.092	--		0.984	0.000 *	0.000 *	0.984				
LSF AI	0.768	0.000 *	0.000 *	0.000 *	0.001 *	0.000 *		0.011 *	0.002 *	0.000 *	0.984	--	0.000 *	0.000 *	0.000 *	0.000 *			0.003 *	0.023 *
Naeser 2		0.000 *	0.984										--	0.000 *	0.000 *					
Olsen (OLCR)														--						
Olsen (standalone)		0.025 *												0.000 *	--					
PEARL-DGS		0.000 *	0.002 *			0.984			0.984	0.638			0.984	0.000 *	0.000 *	--				
SRK/T	0.010 *	0.000 *	0.000 *	0.000 *	0.000 *	0.000 *	0.000 *	0.000 *	0.000 *	0.000 *	0.008 *	0.000 *	0.000 *	0.000 *	0.000 *	0.000 *	--	0.002 *	0.000 *	0.000 *
T2	0.268	0.000 *	0.000 *	0.000 *	0.000 *	0.000 *	0.984	0.005 *	0.002 *	0.002 *	0.206	0.984	0.000 *	0.000 *	0.000 *	0.001 *		--	0.003 *	0.016 *
VRF CMAL		0.000 *	0.003 *	0.014 *	0.322	0.045 *		0.760	0.211	0.003 *	0.984		0.000 *	0.000 *	0.000 *	0.122			--	0.984
VRF-G		0.000 *	0.018 *	0.984	0.984	0.664		0.984	0.984	0.030 *	0.984		0.984	0.000 *	0.000 *	0.984				--

*p*-values = calculated probability. α = 0.05—significance level. * Statistically significant difference with other formulas.

**Table 3:** Multiple comparisons of the formulas according to the percentage of eyes within ±0.50 D of the predicted refraction, according to McNemar’s Chi-squared test with continuity correction, to determine *p* values for every pair of formulas and the adjusted *p* values using Holm’s correction (*p* < 0.05).

Formula:	Different (*p* < 0.05) from Formula:
BUII	Olsen (OLCR)
Castrop	Holladay 1, SRK T
EVO 2.0	Olsen (OLCR)
Haigis	-
Hoffer Q	-
Hoffer QST	Olsen (OLCR)
Holladay 1	Castrop, Olsen (OLCR)
Holladay 2	Olsen (OLCR)
Cooke K6	-
Kane	-
Karmona	Olsen (OLCR)
LSF AI	Olsen (OLCR)
Naeser 2	-
Olsen (OLCR)	BUII, EVO 2.0, Hoffer QST, Holladay 1, Holladay 2, Karmona, LSF AI, PEARL-DGS, SRK/T, T2, VRF CMAL, VRF-G
Olsen (standalone)	-
Pearl-DGS	Olsen (OLCR)
SRK/T	Castrop, Olsen (OLCR)
T2	Olsen (OLCR)
VRF CMAL	Olsen (OLCR)
VRF-G	Olsen (OLCR)

*p*-values = calculated probability. α = 0.05—significance level.

**Table 4:** Refractive outcomes and optimized constants obtained by each formula in medium-long eyes. The mean prediction error (PE), standard deviation of errors (SD), mean absolute error (MAE), median absolute error (MedAE), mean absolute deviation (MAD), optimized constants, and percentage of eyes with refractive prediction errors within ±0.25 D, ±0.50 D, ±0.75 D, and ±1.00 D for each of the 20 formulas. The best standard deviation values (SD) were found for SRK/T (0.179 D), T2 (0.216 D), and Holladay 1 (0.219 D); the worst result was produced by the Olsen (OLCR) formula (0.468 D).

IOL	Alcon IQ SN60WF									
Axial Length	24.50–25.99 mm									
*n*	95									
Formula	Optimized Constants	PE	SD	MAE	MedAE	MAD	Eyes Within PE (%)			
	Alcon IQ SN60WF						PE ≤ 0.25 D	PE ≤ 0.50 D	PE ≤ 0.75 D	PE ≤ 1.00 D
BUII	1.880	0.001	0.259	0.189	0.134	0.190	77.89	93.68	97.89	100.00
Castrop	0.410 0.112	0.000	0.417	0.327	0.249	0.327	50.53	80.00	92.63	98.95
EVO 2.0	119.340	0.000	0.316	0.247	0.203	0.247	57.89	91.58	97.89	100.00
Haigis	−0.817 0.218 0.215	0.000	0.293	0.218	0.161	0.218	69.47	90.53	97.89	100.00
Hoffer Q	5.630	0.000	0.293	0.223	0.175	0.223	66.32	88.42	98.95	100.00
Hoffer QST	5.655	0.000	0.297	0.231	0.211	0.231	58.95	92.63	97.89	100.00
Holladay 1	1.771	0.000	0.219	0.160	0.109	0.160	81.05	96.84	98.95	100.00
Holladay 2	5.624	0.000	0.276	0.204	0.147	0.204	68.42	92.63	97.89	100.00
Cooke K6	118.971	0.000	0.298	0.228	0.183	0.228	63.16	90.53	97.89	100.00
Kane	119.018	0.000	0.317	0.235	0.162	0.235	61.05	89.47	95.79	100.00
Karmona	119.361	−0.001	0.265	0.201	0.151	0.201	72.63	93.68	97.89	100.00
LSF AI	118.919	0.000	0.232	0.161	0.101	0.161	80.00	94.74	100.00	100.00
Naeser 2	1.446 0.943	0.001	0.303	0.220	0.151	0.221	68.42	90.53	96.84	100.00
Olsen (OLCR)	4.735	0.000	0.468	0.366	0.316	0.366	44.21	75.79	89.47	95.79
Olsen (standalone)	4.788	0.000	0.400	0.315	0.292	0.315	48.42	85.26	93.68	98.95
PEARL-DGS	119.056	0.000	0.294	0.231	0.194	0.231	64.21	92.63	97.89	100.00
SRK/T	119.061	0.000	0.179	0.127	0.090	0.127	87.37	98.95	98.95	100.00
T2	118.878	0.000	0.216	0.157	0.113	0.157	83.16	94.74	100.00	100.00
VRF CMAL	5.504	0.000	0.262	0.190	0.147	0.189	74.74	93.68	100.00	100.00
VRF-G	119.144	0.000	0.272	0.207	0.175	0.207	70.53	93.68	98.95	100.00

PE = mean prediction error. SD = standard deviation of the error. MAE = mean absolute error. MedAE = median absolute error. MAD = mean absolute deviation. D = diopter. *n* = number of cases.

## Data Availability

The data presented in this study are available on request from the corresponding author. The data are not publicly available due to privacy.

## Abbreviations

The following abbreviations are used in this manuscript:

ACD	Anterior chamber depth
AI	Artificial intelligence
CCT	Central corneal thickness
ELP	Effective lens position
IOL	Intraocular lens
K	Keratometry
LSF AI	Ladas Super Formula AI
LT	Lens thickness
MAE	Mean absolute error
MedAE	Median absolute error
PE	Prediction error
SD	Standard deviation
WTW	White-to-white
